# A Look Back at the Second Generation of Ochsner Research

**DOI:** 10.31486/toj.24.5052

**Published:** 2024

**Authors:** Richard N. Re

**Affiliations:** Vice President of Research and Chief Scientific Officer (retired), Ochsner Clinic Foundation, New Orleans, LA

*Editor's Note: Dr Richard Re, who retired in summer 2024, had a long and distinguished research career at Ochsner. Recruited from Massachusetts General Hospital, Harvard Medical School, Dr Re joined Ochsner in 1979 and was instrumental in developing Ochsner's strong translational research program whose scientists produced groundbreaking work in molecular immunology, molecular oncology, and molecular genetics. During his career, Dr Re published more than 185 peer-reviewed papers and coauthored several books and book chapters. For the past several years, he has served as Chief Scientific Officer for Ochsner Health. On the occasion of his retirement, Dr Re agreed to write this historic overview of translational research activity at Ochsner. As well as documenting important discoveries by Ochsner scientists, this overview serves as a tribute to Dr Re's vision and his work.*

**Figure.**
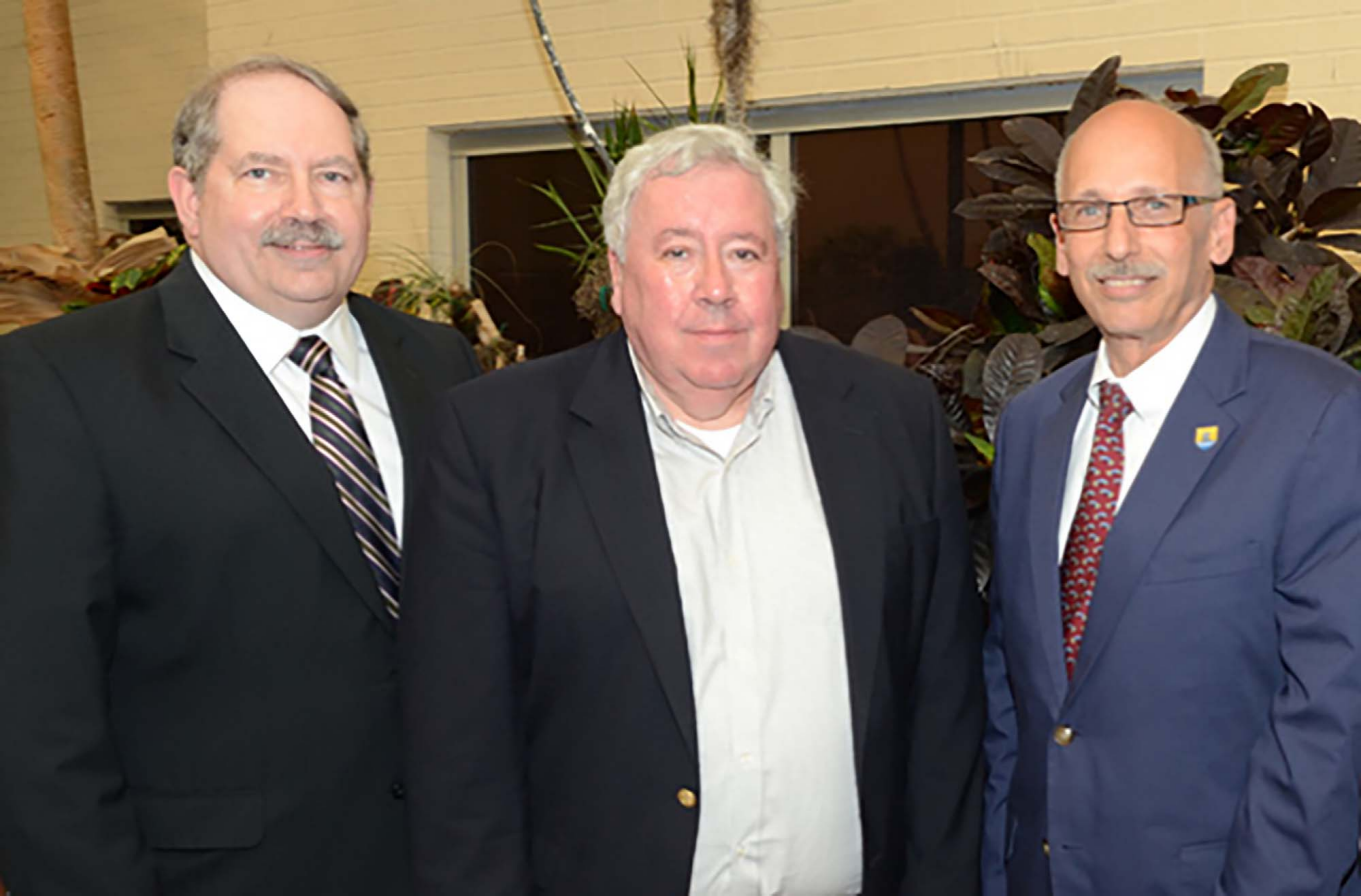
Richard N. Re, MD (center), former Vice President of Research and Chief Scientific Officer, is pictured at Ochsner Research Night 2011 with Joseph Bisordi, MD (left), former Executive Vice President and Chief Medical Officer, and William Pinsky, MD (right), former Executive Vice President and Chief Academic Officer.

## EARLY RESEARCH PIONEERS

The Ochsner Clinic and the Alton Ochsner Medical Foundation were established in the early 1940s as what was then a new mode of medical practice—the academic group practice. The five founders were academic physicians from Tulane University who valued research and education and wanted to instill those activities in their new organization. Indeed, they fully expected that once the value of their group practice model was widely appreciated, the clinic and foundation would merge with Tulane. Their commitment to research manifested early when they recruited Dr Otto and Mrs Selma Schales from the Peter Bent Brigham Hospital, Harvard Medical School, to conduct research on the blood pressure–raising enzyme renin. This expansion involved establishing a research laboratory near the first clinic building on Prytania Street. Later, Dr Albert Segaloff was recruited from Wayne State to head an oncology research effort. Segaloff became a giant in oncology, especially in the area of hormonal influences on breast cancer. He established and edited the journal *Steroids*, and he oversaw the construction of the Freeman Research Building at Ochsner's Jefferson Highway campus. Next, Edward Frohlich, a nationally recognized expert in hypertension research, was recruited from the University of Oklahoma. Frohlich not only studied circulatory pathophysiology in rat models, but he was also a pioneer in the study of the hemodynamics of various forms of hypertension in patients. His work complemented the clinical trials that were ongoing throughout the clinic departments. In time, the American Heart Association named Frohlich editor of its journal *Hypertension.* The story of Ochsner research to this point, including Alton Ochsner's important work on smoking as a cause of lung cancer, is well described in several works about the clinic and foundation.^1-4^

## THE SECOND GENERATION

At the time Frohlich joined Ochsner, I was at Massachusetts General Hospital, Harvard Medical School, working primarily in clinical endocrine research. Much of this work was in the area of hypertension, and I became interested in the biology of the blood pressure–raising hormone angiotensin II. The idea of joining a new effort in hypertension research appealed to me. I joined the Ochsner Clinic endocrine section and set up a laboratory in the Freeman Research Building. In 1986, I became vice president of research with a mandate to provide research support to all clinical departments. Because molecular genetics and cellular immunology were rapidly emerging as powerful new tools applicable to many disciplines, I enlisted the support of Dr George Porter, then the president of the Alton Ochsner Medical Foundation, to launch an initiative to bring those disciplines to Ochsner. Dr Porter had trained at both the National Institutes of Health (NIH) and the Peter Bent Brigham Hospital, and he appreciated the value such an initiative could bring to the organization. A major philanthropic drive was undertaken to build new laboratories and recruit scientists who could perform first-rate research and whose work would potentially be widely applicable. This initiative was featured in the journal *Molecular Medicine*.^5^

I also enlisted Dr Porter's support to launch a major effort in health service outcomes research, recruiting Dr. Marie Krousel-Wood to the project; that initiative, although successful on all levels, is sufficiently different from the basic science program that it is best left to another review.

### Laboratory of Molecular Immunology

Dr Yong-Sun Choi was recruited from Sloan Kettering Institute in New York to establish the Laboratory of Molecular Immunology. His lab came to include Drs Li Li and Xin Zhang, who remain scientifically productive at Ochsner today, and together they generated many important research findings. This group demonstrated that the 8D6 Ag surface antigen on lymph node follicular dendritic cells, later called CD320, stimulated the proliferation of B lymphocytes. They went on to investigate the possible role of this molecule in lymphoma. Early on, the Choi lab established a program of adoptive immunotherapy for cancer. This therapy, which originated in laboratories at the NIH, involved culturing T lymphocytes from cancer patients with interleukin 2 and then reinfusing them back into the patient to mount an immunologic attack on the cancer. Patients came to Ochsner from around the country for this treatment. Dr John Bolton oversaw their therapy in a specially outfitted intensive care unit at Ochsner Medical Center. Some significant positive results were obtained, but based on results obtained nationwide, the program was discontinued for lack of benefit in a sufficient number of patients to make it a viable therapeutic option. The therapy did, however, serve as the precursor for CAR T-cell therapy that is widely used today and involves the reinfusion of T cells after they have been molecularly engineered to attack the patient's tumor. The Choi group produced other important results, including better defining the role of stromal cells in cancer. They obtained significant NIH funding over the years and were once featured on the cover of the journal *Immunology.*

### Laboratory of Molecular Oncology

Dr Om Prakash was recruited from Columbia University in New York to establish the Laboratory of Molecular Oncology. He studied the molecular pathology of neuroblastoma, Kaposi sarcoma, and leukemia, performing molecular and cytogenetic analyses on B-cell chronic lymphocytic leukemia. He also established a transgenic laboratory to facilitate his studies and those of other investigators. In those days, Dr Prakash's lab was one of only two such laboratories in Louisiana, the other being at Louisiana State University (LSU) in Baton Rouge. A major effort of the laboratory was the elucidation of the role of the Tat protein in HIV-produced diseases, a pressing issue during the height of the AIDS epidemic.

### Laboratory of Molecular Genetics

To establish the Laboratory of Molecular Genetics, we enlisted the help of Dr Prescott Deininger, then of LSU Medical Center. He recommended the husband and wife team of Dr Julia Cook and Dr Jawed Alam. At Ochsner, Alam did forefront work on the response to oxidative stress and the molecular biology of heme oxygenase, working in collaboration with scientists at Yale and elsewhere. Cook focused originally on cancer; in particular, she investigated the role of normal and mutant p53 tumor suppressor proteins. She and I found that oligonucleotides designed to bind in a triplex fashion to a specific p53 binding site homology suppressed the growth of various cancer cells in culture.

My interest, however, focused on the intracellular actions of peptide hormones. This kind of intracellular action, which I termed “intracrine,” was not recognized in those days, but because I had already shown that the peptide hormone angiotensin II binds to nuclei and upregulates gene transcription, and, with the late Sara Bryan of the University of New Orleans, had demonstrated chromatin binding of the peptide with changes indicative of gene transcription, Cook saw value in the idea and accepted my request to join me in the work. Her research helped establish and define the intracellular actions of the peptide hormone angiotensin II. This work led to numerous publications in major journals and to NIH funding. The details of angiotensin trafficking from the extracellular space to the cell nucleus, delineation of the second messengers activated by intracellular angiotensin II, the pathologic role of angiotensin receptor (AT1R) fragments, the regulation of AT1R intracellular trafficking to the cell surface by a previously unrecognized associated binding protein (GABARAP), and the demonstration that the downregulation of that binding protein resulted in blood pressure lowering in hypertensive animals were important results of this work. Cook designed a DNA construct encoding an intracellular fluorescent angiotensin II fusion protein that we not only used in a transgenic mouse model to demonstrate the pathologic effects of overexpression of intracellular angiotensin II, but that she also made freely available to hypertension investigators around the country who, in turn, used it to make important breakthroughs such as demonstrating the role of renal tubular intracellular angiotensin II in hypertension. In many of these studies, Alam pitched in to help and thereby round out our group.

Other work in molecular medicine involved the development of novel polymerase chain reaction assays for disease diagnosis (Dr Sonia Montenegro James) and the study of micro RNAs as indicators of carotid artery plaque rupture (Drs Cooper Woods and Hernan Bazan). This work was done in addition to studies of novel percutaneous techniques for the treatment of coronary artery disease (Drs Stephen Ramee and Christopher White), internationally recognized physiologic work on hypertension (Drs Ed Frohlich, Franz Messerli, and Hector Ventura), basic studies of infectious disease treatment (Dr Kin Panky), and studies of the role of the cystic fibrosis transporter in disease and in lung development (Dr Janet Larson), as well as many translational collaborations between clinicians and these laboratories.

## FOCUS ON CLINICAL TRIALS RESEARCH

It was an exciting period. In time, however, management made the decision to focus on clinical trial research, and instead of growing an Ochsner basic science base, elected to use collaborations with established academic partners such as the University of Queensland and LSU Shreveport to provide basic science/translational support for the Ochsner research effort. This approach was not foreign to the organization in that the founders wanted to develop strong basic research but with an eye to joining Tulane, a merger that never came to pass. The current program has worked well, and Ochsner research is growing and productive. But for those of us who participated in the excitement of those early days, there remains more than a touch of nostalgia in thinking back over that time.
